# Delegates to Medical Convention in London

**Published:** 1881-08

**Authors:** 


Dr. George J. Friedricks, Second Vice-President of the American Dental association, another of our subscribers and friends from New Orleans, La., and a writer for our columns, sailed for Europe after the adjournment, a delegate to the Medical Convention in London, to be held in this month, August. He promised us a line from there. He has been preceded by Dr. Austin Flint, of NewYork, and Drs. Harlan and Dean, of Chicago, and was accompanied by Dr. Wm. H. Atkinson, Dr. E. P. Brown, Dr. I. F. Wardwell and Dr. C. S. Wardwell, of New York, Dr. C. R. Butler, of Cleveland, Dr. J. Taft, of Cincinnati, Dr. Webb, of Philadelphia, and others.

